# Vanishing left ventricular thrombus in a woman with peripartum cardiomyopathy: a case report

**DOI:** 10.1186/1756-0500-5-544

**Published:** 2012-10-02

**Authors:** Wafa A Altuwaijri, Iain DC Kirkpatrick, Davinder S Jassal, Anita Soni

**Affiliations:** 1 Department of Internal Medicine, University of Manitoba, Winnipeg, Manitoba, Canada; 2 Department of Radiology, St. Boniface General Hospital, University of Manitoba, Winnipeg, Manitoba, Canada; 3 Section of Cardiology, Department of Internal Medicine, Faculty of Medicine, University of Manitoba, 409 Taché Avenue, Winnipeg, R2H 2A6, Manitoba, Canada; 4 Institute of Cardiovascular Sciences, Department of Physiology, St. Boniface General Hospital, University of Manitoba, Winnipeg, Manitoba, Canada; 5 Cardiology, Radiology and Physiology, Bergen Cardiac Care Centre, St. Boniface General Hospital, Winnipeg, R2H 2A6, Canada

**Keywords:** Peripartum cardiomyopathy, Multimodality cardiac imaging, Thrombus

## Abstract

**Background:**

Peripartum cardiomyopathy (PPCM) is a rare cardiac disorder characterized by the development of heart failure in the last month of pregnancy or up to 5 months postpartum in women without other identifiable causes of cardiac failure. The combination of left ventricular (LV) systolic dysfunction and hypercoaguability can cause thromboembolic complications including intra-cardiac thrombi.

**Case presentation:**

A 25-year-old Caucasian female with PPCM demonstrated multiple thrombi in the LV on transthoracic echocardiography. Following anticoagulation with parenteral heparin, a cardiac MRI four days later demonstrated near resolution of the thrombi.

**Conclusion:**

We review the presentation, diagnosis and management of LV thrombi in the clinical setting of PPCM.

## Background

Peripartum cardiomyopathy (PPCM) is a rare cardiac disorder characterized by the development of heart failure in the last month of pregnancy or up to 5 months postpartum in women without other identifiable causes of cardiac failure. The combination of left ventricular (LV) systolic dysfunction and hypercoaguability can cause thromboembolic complications including intra-cardiac thrombi. Although there are two case reports of PPCM with rapid resolution of intracardiac thrombi diagnosed by transthoracic echocardiography (TTE) in the literature to date, our case illustrates the use of multimodality cardiac imaging including TTE and cardiac MRI (CMR) in this patient population. We review the presentation, diagnosis and management of LV thrombi in the clinical setting of PPCM.

## Case presentation

**Figure 1:**
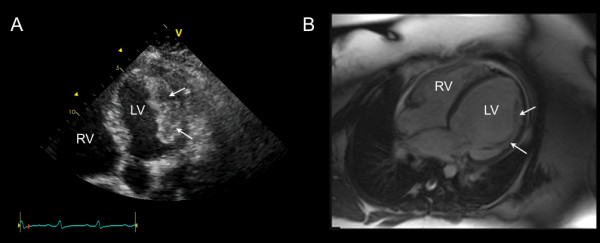
**A) An apical 5 chamber view on TTE demonstrating a large layered echodense mass attached to the lateral wall of the LV consistent with thrombi. B**) Four-chamber view of a balanced steady-state free precession CMR acquisition demonstrating that the thrombus has nearly completely resolved, leaving behind only the underlying muscular trabeculations on this view (arrows).

A 25-year-old G4, P4 Caucasian female presented with a history of worsening dyspnea, orthopnea and paroxysmal nocturnal dyspnea seven months post-partum. On physical examination, she was tachycardic with a heart rate of 110 bpm and a normal blood pressure of 110/70 mmHg. The heart sounds were within normal limits with decreased breath sounds bilaterally and lower limb edema. Initial blood work including the complete blood count, electrolytes, liver function tests and cardiac biomarkers were within normal limits. The electocardiogram demonstrated sinus tachycardia and the chest x-ray confirmed cardiomegaly with pulmonary venous congestion. Transthoracic echocardiography (TTE) demonstrated biventricular dilation, biatrial enlargement, global hypokinesis of the left ventricle (LV) with an ejection fraction less than 20%. There were multiple echodense masses within the LV, consistent with thrombi (Figure 1A). With a diagnosis of peripartum cardiomyopathy (PPCM), the patient was appropriately started on diuretics, beta blockade, ACE inhibition and parenteral heparin for the LV thrombi. A subsequent cardiac MRI (CMR) 4 days later confirmed severe biventricular systolic dysfunction, with significant resolution of the thrombi previously detected on TTE (Figure 1B).

## Discussion

Peripartum cardiomyopathy (PPCM) is a disorder of unknown cause in which LV ventricular systolic dysfunction and symptoms of heart failure occur between the last month of pregnancy and the first 5 months postpartum. The incidence of PPCM varies from the infrequent one case per 4000 live births in the US to the more frequent one case per 1000 live births in South Africa or one case per 299 live births in Haiti [1]. Peripartum cardiomyopathy has been associated with several risk factors including increased age, gravidity or parity, African origin, toxaemia or hypertension of pregnancy, use of tocolytics, twin pregnancy, obesity and low socioeconomic status [1]. A number of potential etiologies for PPCM have been proposed including myocarditis, abnormal immune response to pregnancy, maladaptive response to the hemodynamic stresses of pregnancy, stress-activated cytokines, and prolonged tocolysis [1]. As a diagnosis of exclusion, women with PPCM are treated with similar medications as the general heart failure population including beta blockers, nitrates, hydralazine and digoxin [1].

Women with PPCM are at high risk for thrombus formation and thromboembolism due to both the hypercoagulable state of pregnancy and stasis of blood due to severe LV systolic dysfunction. Although two-dimensional TTE is the most commonly used technique for the non-invasive identification and follow-up of LV thrombus, the recent introduction of contrast echo and cardiac MRI (CMR) may improve thrombus detection. In a recent study evaluating 361 patients, 2D TTE was compared to both TEE and CMR for the identification of LVT. Although TTE had a sensitivity and specificity of 23±12% and 96±4% for the diagnosis of LVT, CMR had a higher sensitivity and specificity of 88±9% and 99±2%, respectively [2]. In a different study, although the administration of LV opacification during TTE doubled sensitivity and improved accuracy for the detection of LVT, 9 out of 23 (39%) thrombi were still missed as compared to delayed enhancement CMR imaging [3].

In women with PPCM, LV thrombus (LVT) is detected in up to 17% of patients, in whom the risk of systemic embolization is high [4]. A review of the literature demonstrated only two echocardiographic cases of rapid resolution of LVT during PPCM following parenteral anticoagulation [5,6]. Kim DY et al. described a 22-year-old female with a PPCM and multiple biventricular thrombi on TTE that resolved following 3 weeks of anticoagulation [5]. Nishi I et al. described a similar case of a 23-year-old with PPCM and biventricular thrombi on TTE that resolved rapidly after 4 days of parenteral anticoagulation [6]. Our case illustrates the use of multimodality cardiac imaging including TTE and CMR for both the detection and rapid resolution of LVT in this patient population. We recommend a minimum of 6 months of anticoagulation to prevent life threatening embolization in women with PPCM.

## Conclusion

The use of multimodality cardiac imaging including TTE and CMR can provide complementary information for the management of LVT in the clinical setting of a PPCM.

## Consent

Written informed consent was obtained from the patient for publication of this case report and accompanying images. A copy of the written consent is available for review by the Editor-in-Chief of this journal.

## Competing interests

The authors declare that they have no competing interests.

## Authors’ contributions

WA, DJ, IK and AS contributed to the writing of the manuscript. All authors read and approved the final manuscript.
